# Condition-Dependent Sex Differences in Lumbar sEMG and Prefrontal Hemodynamics During Known-Weight and Weight-Misprediction Lifting: A Pilot Study

**DOI:** 10.3390/bioengineering13080871

**Published:** 2026-07-28

**Authors:** Jaeyoung Shin, Jae-Ho Han

**Affiliations:** 1 Department of AI Data Engineering, Korea National University of Transportation, Uiwang 16106, Republic of Korea; 2 Department of Brain and Cognitive Engineering, Korea University, Seoul 02841, Republic of Korea; 3 Department of Precision Public Health, Korea University, Seoul 02841, Republic of Korea

**Keywords:** object lifting, weight uncertainty, physical activity level, surface electromyography, functional near-infrared spectroscopy

## Abstract

This pilot study examined sex-related neuromuscular and prefrontal hemodynamic responses across 3 and 9 kg known-weight and weight-misprediction lifting conditions and explored whether these patterns differed by physical activity level (PAL). Thirty adults completed the lifting tasks. Among the unknown-weight trials, only wrong-guess trials were retained, while the known-weight trials served as comparison conditions. The integrated linear mixed-effects model showed a significant Condition × Sex interaction, indicating that sex-related differences varied across lifting conditions. The omnibus condition and sex effects were also significant, whereas PAL and all interactions involving PAL were not significant. Exploratory PAL-stratified analyses showed condition-dependent sex differences within both PAL groups. In the moderate PAL group, women showed higher task-related ΔHbO β-values than men across multiple channels under the 3 kg wrong-guess condition after false discovery rate correction. These preliminary findings suggest condition-dependent sex-related neuromuscular responses and sex-related differences in prefrontal hemodynamic responses observed under the 3 kg wrong-guess condition. The exploratory subgroup findings require confirmation in larger and more balanced samples.

## 1. Introduction

Lifting objects is a common motor task in both industrial and daily life. In particular, when the weight of an object is unknown before lifting, the predicted weight may differ from the actual weight. Such weight-misprediction can lead to inappropriate lifting strategies and increase the risk of musculoskeletal injury [1]. In these situations, rapid changes in trunk muscle activation and lumbar spine kinematics have been reported, which tend to increase external lumbar loading [2,3]. Recent biomechanical studies have further characterized spinal loading and low-back exposure across different lifting strategies, unstable load conditions, and person-specific risk profiles [1,2,3,4,5]. Video-based methods have also been validated for quantifying whole-body lifting strategy [6]. Furthermore, from a neuroengineering perspective, lifting objects represents a complex motor control process that integrates sensory feedback, motor planning, and attentional control, with the prefrontal cortex (PFC) playing a key role in this control function [4,5]. Recent fNIRS studies have examined prefrontal responses in relation to habitual physical activity [7] and executive demands during motor tasks such as walking [8,9].

In particular, when weight information is uncertain during object lifting, continuous motor control is required to maintain movement accuracy and safety, which may increase the recruitment of cognitive resources in the PFC. Task-related cognitive involvement can also alter kinematic and neuromuscular control during movement [10]. Previous studies have shown that lifting unknown loads can alter both peripheral muscle activity and brain responses [6]. In addition, Cheng et al. examined sex- and physical activity-related characteristics associated with lumbar paraspinal muscle activation [7], while Martinez et al. reported sex-related differences in muscle activation and estimated muscle forces during box lifting [8]. Recent evidence further indicates that sex, strength capacity, fatigue, and other structural or functional personal factors can influence lifting biomechanics and low-back exposure [11,12,13]. These findings indicate that sex and physical activity-related characteristics may contribute to variability in neuromuscular responses; however, their combined influence during weight-uncertain lifting remains insufficiently understood. Combined EMG–NIRS approaches have been used to assess the electrical and hemodynamic activity of working muscles [9,14], while simultaneous sEMG–fNIRS has been used to examine peripheral muscle activation together with cortical dynamics during motor tasks [10,15,16]. More broadly, multimodal physiological signals have been used to characterize and predict cognitive load [17]. However, to the best of our knowledge, lifting under weight-misprediction conditions has not been investigated using simultaneous sEMG and fNIRS with consideration of both sex and PAL.

Given this gap, we simultaneously measured lumbar sEMG and prefrontal fNIRS signals during an object-lifting task. Accordingly, this pilot study aimed to characterize lumbar muscle and prefrontal hemodynamic responses across 3 and 9 kg lifting trials under known-weight and weight-misprediction conditions and to explore whether sex-related response patterns differed by PAL.

## 2. Materials and Methods

Based on an a priori power analysis conducted using G*Power 3.1.9.7, the required sample size was estimated to be 30 to detect the overall condition effect in the repeated-measures design. A sensitivity analysis for two-sided between-sex comparisons indicated minimum detectable effect sizes of Cohen’s d = 1.56 and 1.70 for the high and moderate PAL groups, respectively, at a nominal α = 0.05 and 80% power. Given these relatively large minimum detectable effect sizes, the PAL-stratified subgroup analyses were considered exploratory. The channel-wise analyses were also treated as exploratory because of the limited subgroup sample sizes and the multiple-comparison burden. PAL was assessed using the International Physical Activity Questionnaire Short Form, Last 7 Days, Self-Administered Format (IPAQ-SF), and participants were classified into moderate and high PAL groups according to the standard IPAQ scoring protocol. The high PAL group consisted of ten men and six women, whereas the moderate PAL group consisted of five men and nine women.

**Figure 1:**
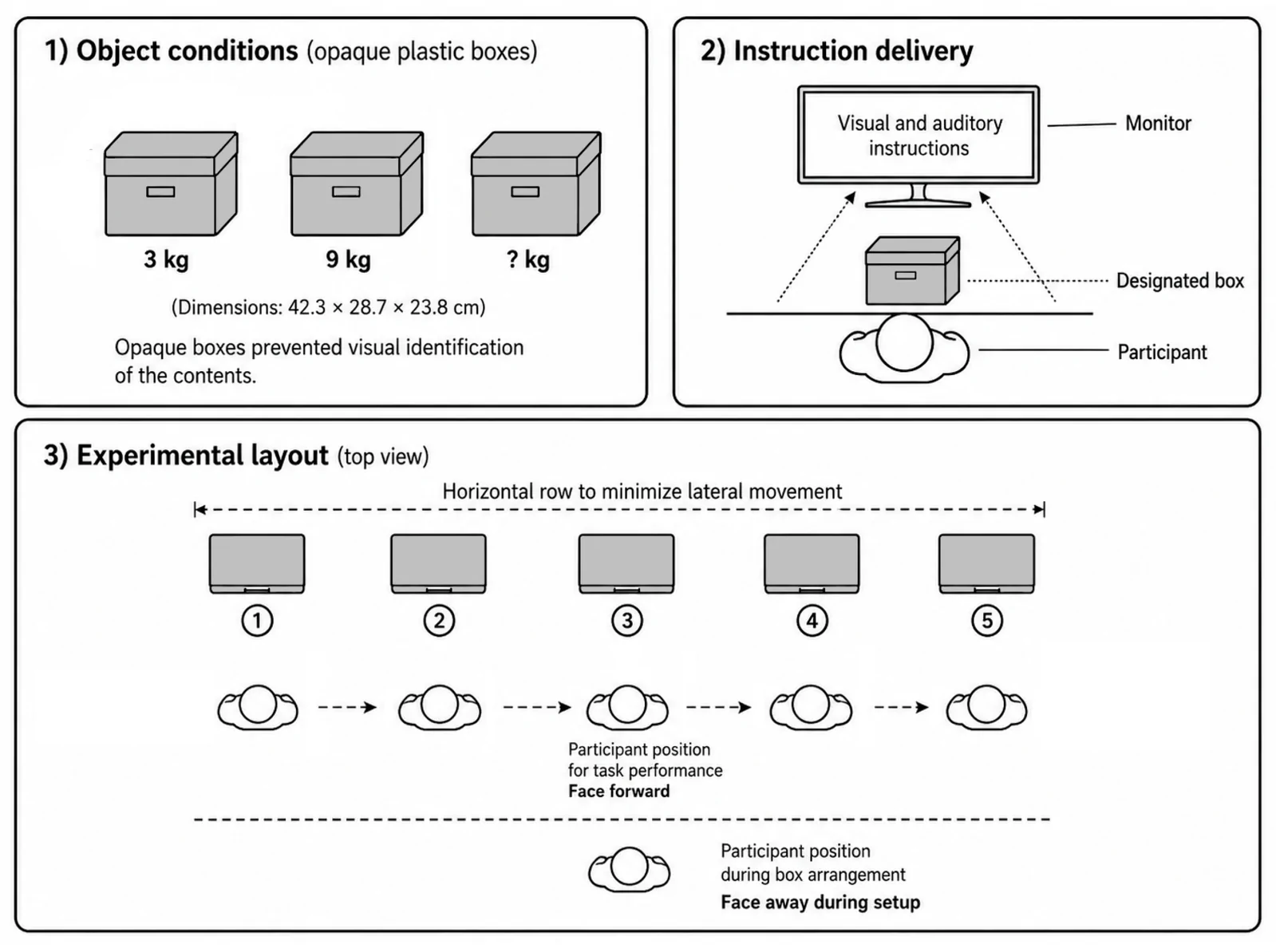
Experimental setup for object lifting task.

Manual muscle tests were conducted before the main experiment and the maximal voluntary contraction (MVC) obtained from three repeated measurements was used as the reference value for normalization. The main experiment was conducted across six sessions, with four conditions defined by whether participants were given prior information on the object weight (known, KNW, or unknown, UKN) they lifted and by the actual object weights (3 or 9 kg). When the weight information was provided in advance, the experiment was conducted in one session per weight; otherwise, it was conducted in two sessions per condition. Each session included five blocks of lift-release tasks. Therefore, the participants performed 30 lift–release tasks (6 sessions × 5 blocks). The order of sessions for each condition was counterbalanced to minimize order effects. The lifting task was performed using opaque plastic boxes (42.3 × 28.7 × 23.8 cm) to prevent participants from visually identifying their contents [11]. The experimental setup is shown in Figure 1. Before each session, participants remained seated facing away from the experimental setup while the boxes were arranged. They then stood in front of the designated box and followed visual and auditory instructions presented on a 24-inch monitor using E-Prime 3.0 (Psychology Software Tools, Inc., Pittsburgh, PA, USA). The boxes were positioned in a horizontal row to minimize lateral movement. Each trial consisted of 2 s of instruction, 15 s of lifting the box to a comfortable standing position with the arms extended downward and holding it in that position, and 30 s of rest in a standing A-pose with the arms slightly abducted from the trunk. A 2 min rest period was provided between sessions. Before data collection, participants completed a practice session to standardize lifting posture and movement rhythm. In the UKN condition, participants were asked to guess the weight of the object immediately before lifting.

**Figure 2:**
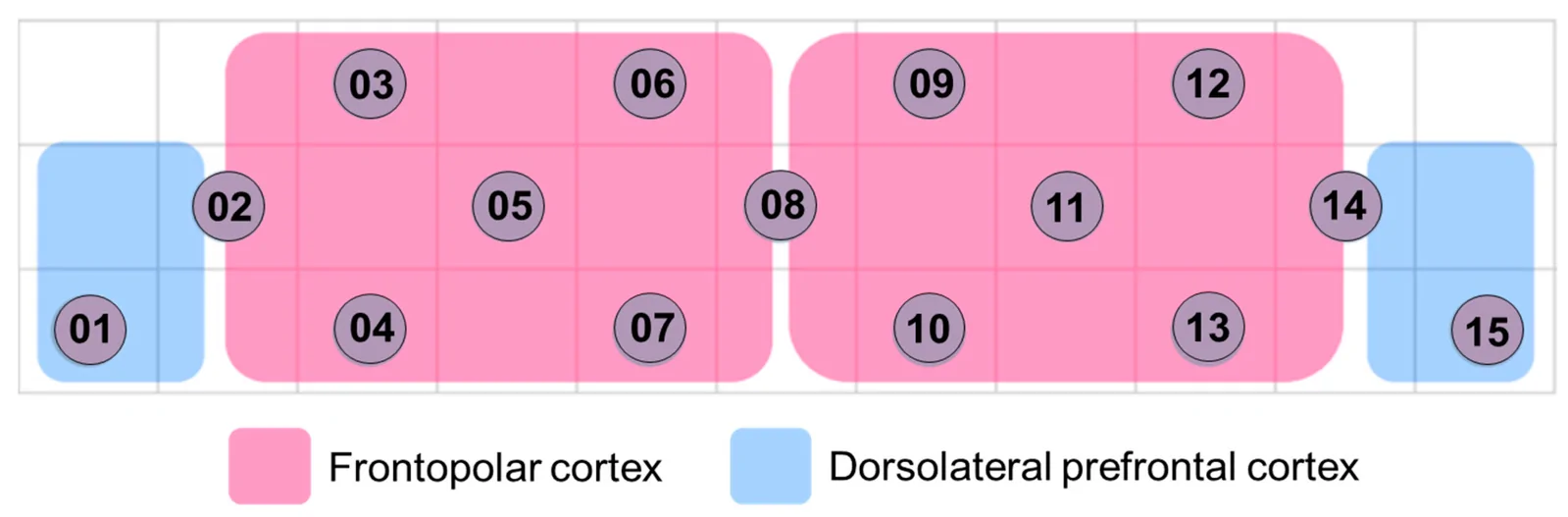
fNIRS channel locations.

To measure muscle activity during object lifting, a two-channel wireless sEMG device (Delsys Inc., Natick, MA, USA) was attached to the L3 erector spinae muscles on both sides, and the signals were collected at a sampling rate of 2000 Hz. To measure prefrontal hemodynamic responses, a 15-channel fNIRS device (NIRSIT LITE; OBELAB, Seoul, Republic of Korea) was positioned on the forehead. The channel locations are illustrated in Figure 2. The fNIRS signals were acquired at a sampling rate of 8.135 Hz. Body movements during object lifting were recorded using a built-in gyroscope sensor in the fNIRS device. The sEMG and fNIRS signals were temporally synchronized, and the synchronized signals were used in subsequent analyses.

For efficient analysis, raw sEMG signals were down-sampled to 200 Hz and full-wave rectified. A fourth-order zero-phase Butterworth low-pass filter with a cutoff frequency of 10 Hz was then applied to obtain the muscle activity envelope. The envelope was normalized to the MVC reference obtained from three repeated measurements and expressed as a percentage of MVC. RMS amplitudes were subsequently calculated using a sliding window (window length: 0.2 s; overlap: 0.1 s). RMS values exceeding the predefined threshold of 50% MVC were treated as outliers and replaced using cubic spline interpolation. Finally, the mean normalized RMS amplitude during the predefined 4–6 s interval after task onset was extracted for analysis. For each participant and experimental condition, normalized RMS amplitudes were averaged across the left and right L3 erector spinae muscles and across the retained trials.

After conversion of raw optical intensities measured by the fNIRS device to optical density and subsequently to ΔHbO and ΔHbR using the modified Beer–Lambert law, only the ΔHbO signals were retained for further analysis. First, a fourth-order zero-phase Butterworth band-pass filter (0.01−0.05 Hz) was applied to reduce low-frequency drift and physiological noise. Second, motion artifacts were corrected using linear regression with the gyroscope signal as a covariate. The corrected ΔHbO signals were then block-averaged ($\bar{Y})$, and channel-wise β-values were estimated using a GLM with the canonical hemodynamic response function (X) for each channel, thereby quantifying task-related prefrontal hemodynamic responses [12].

(1) $$\bar{Y}=X\beta +\epsilon$$

(2) $$\beta ={\left(X^{T}X\right)}^{-1}X^{T}\bar{Y}$$

All statistical analyses were performed in Python (3.14.3) using NumPy (2.4.6), pandas (3.0.3), SciPy (1.17.1), and statsmodels (0.14.6). In the statistical analyses, UKN trials were classified as correct- or wrong-guess trials according to whether the guessed and actual object weights matched. Because the analysis focused on weight misprediction, correct-guess trials were excluded. For each participant, the retained wrong-guess trials within each UKN weight condition were averaged to obtain participant-level condition estimates. Thus, the participant, rather than the individual trial, was the unit of statistical analysis. All 30 participants had at least one retained wrong-guess trial within each UKN weight condition and therefore contributed analyzable participant-level estimates for all four conditions. No participant was excluded because of a lack of retained wrong-guess trials. Normalized sEMG was analyzed using a linear mixed-effects model fitted by maximum likelihood. Condition, sex, PAL, and their interactions were entered as fixed effects using sum-to-zero contrasts, while participant-specific intercepts were included as random effects. Omnibus tests of fixed effects were conducted using joint Wald $\chi^{2}$ tests. Model-based sex contrasts were estimated within each PAL group and lifting condition, and Benjamini–Hochberg FDR correction was applied across the four condition-specific contrasts separately within each PAL group. To directly assess whether PAL moderated the sex-related differences, condition-specific contrasts compared the female–male difference between the moderate and high PAL groups. Benjamini–Hochberg FDR correction was applied across the four condition-specific moderation contrasts. As a sensitivity analysis, a generalized estimating equation (GEE) model with a Gaussian distribution, an exchangeable working correlation structure, and robust sandwich covariance estimation was fitted using the same fixed-effects structure as the primary linear mixed-effects model, with participant ID specified as the clustering variable. Additional PAL-stratified models were conducted as exploratory secondary analyses. For the fNIRS analysis, channel-wise sex differences in ΔHbO β-values were assessed using Welch’s independent-samples t-tests to accommodate unequal group sizes without assuming equal variances. The Benjamini–Hochberg FDR correction was applied separately across the 15 channels within each condition and PAL group. FDR-adjusted p-values are reported as q-values. Hedges’ g was calculated to quantify the magnitude and direction of sex-related differences. Statistical significance was defined as *p* < 0.05, with *q* < 0.05 used for FDR-corrected analyses.

## 3. Results

**Figure 3:**
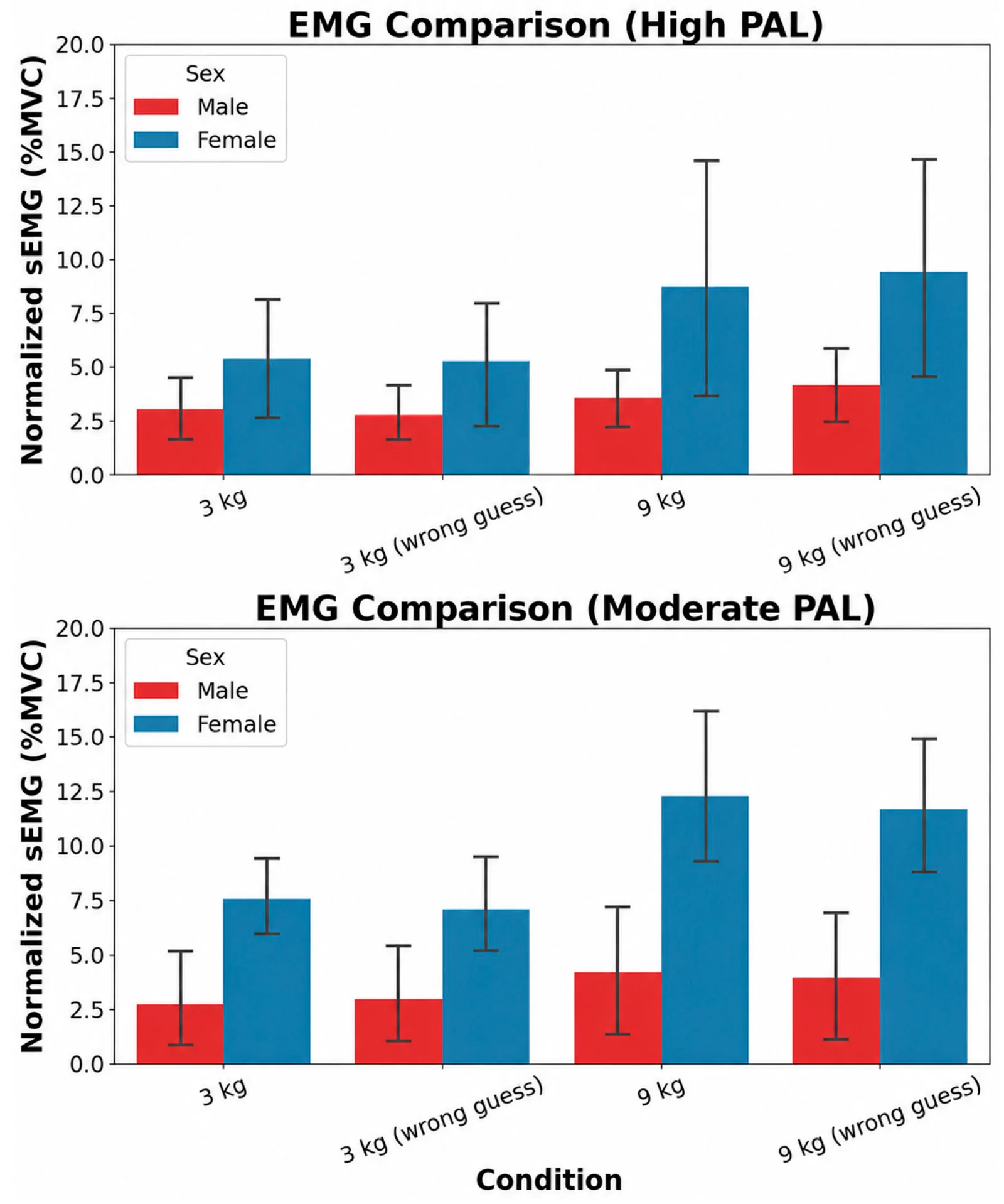
Normalized sEMG activity by sex across lifting conditions within the high and moderate PAL groups. Bars represent mean normalized sEMG activity, and error bars indicate 95% confidence intervals.

Figure 3 presents normalized sEMG activity by condition and sex, and Table 1 presents the results of the integrated linear mixed-effects model. The model showed a significant Condition × Sex interaction ($\chi^{2}$ = 24.282, df = 3, p < 0.001), indicating that sex-related differences in normalized sEMG activity varied across lifting conditions. The omnibus condition and sex effects were also significant, whereas PAL (p = 0.307), Condition × PAL (p = 0.513), Sex × PAL (p = 0.357), and Condition × Sex × PAL (p = 0.937) were not significant. A robust GEE sensitivity analysis yielded the same overall pattern, including a significant Condition × Sex interaction (p = 0.003) and nonsignificant PAL-related interactions.

In model-based comparisons, women in the high PAL group showed significantly higher normalized sEMG activity than men under the 9 kg KNW and 9 kg wrong-guess conditions after FDR correction (both q = 0.015). In the moderate PAL group, women showed significantly higher activity under the 3 kg KNW (q = 0.027), 3 kg wrong-guess (q = 0.041), 9 kg KNW (q < 0.001), and 9 kg wrong-guess conditions (q < 0.001). Direct comparisons of the sex differences between the high and moderate PAL groups were not significant in any condition after FDR correction, indicating that the PAL groups did not significantly differ in the magnitude of the sex-related contrasts. Exploratory PAL-stratified models showed significant Condition × Sex interactions in both the high PAL group ($\chi^{2}$ = 9.391, df = 3, p = 0.025) and the moderate PAL group ($\chi^{2}$ = 16.419, df = 3, p < 0.001).

**Figure 4:**
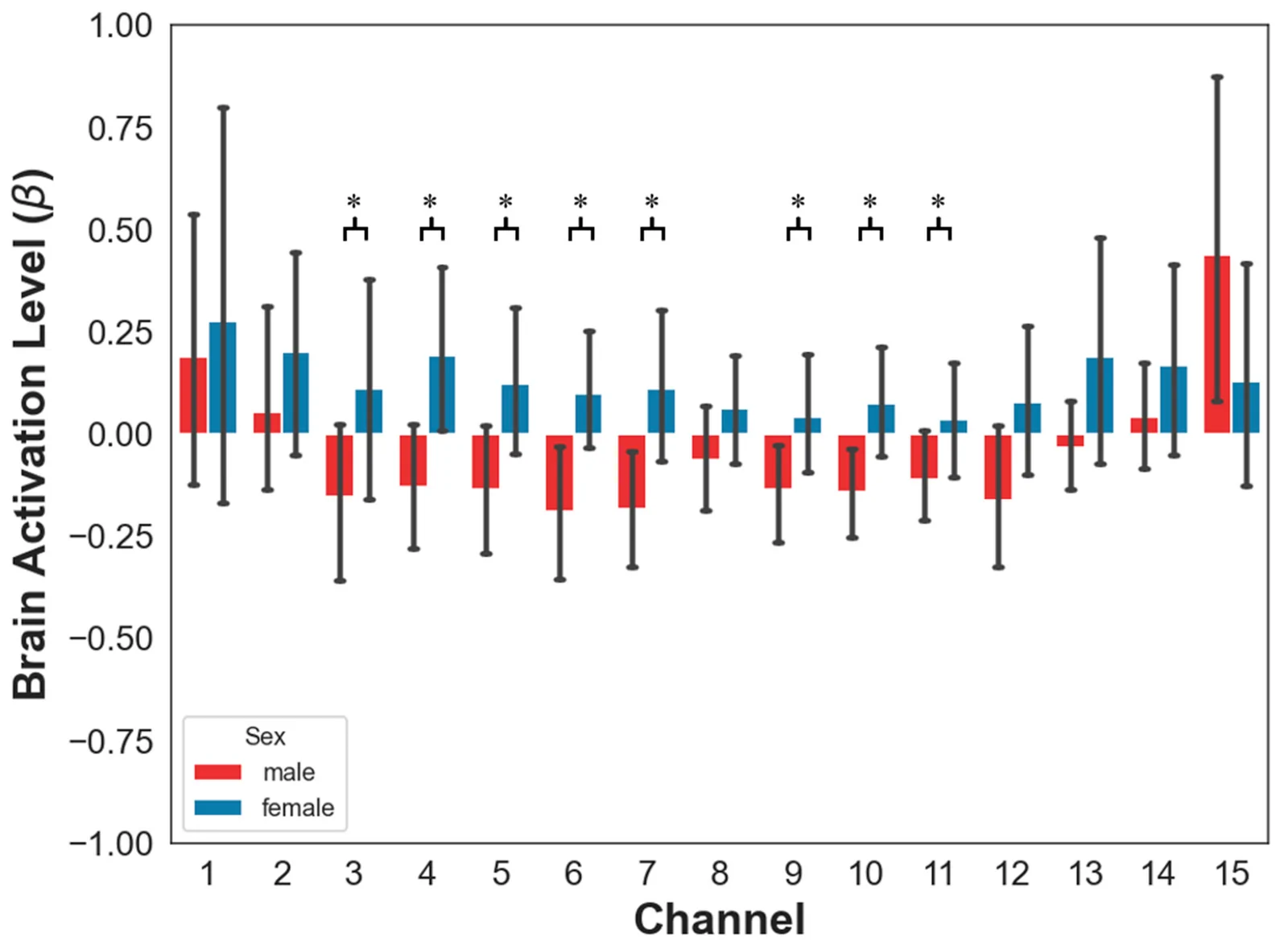
Channel-wise ΔHbO β-values under the 3 kg UKN wrong-guess condition in the moderate PAL group. Asterisks indicate channels showing significant sex-related differences after FDR correction (q < 0.05). Error bars indicate 95% confidence intervals.

Figure 4 and Table 2 present the detailed channel-wise fNIRS results for the moderate PAL group under the 3 kg UKN wrong-guess condition. Complete channel-wise results for all four conditions in both PAL groups, including nonsignificant findings, are provided in Supplementary Table S1. In the moderate PAL group, women showed significantly higher β-values than men across multiple prefrontal channels (Ch03–07 and Ch09–11), as summarized in Table 2. The corresponding FDR-adjusted p-values (q-values) ranged from 0.002 to 0.028, indicating statistically significant sex-related differences after correction for multiple comparisons. Absolute Hedges’ g values ranged from 1.41 to 2.61, indicating large effect sizes. Thus, exploratory channel-wise analyses identified FDR-significant sex-related differences in task-related ΔHbO β-values under the 3 kg weight-misprediction condition.

## 4. Discussion

The integrated linear mixed-effects model demonstrated a significant Condition × Sex interaction, indicating that sex-related differences in normalized sEMG activity varied across lifting conditions. Women showed significantly higher normalized sEMG activity than men under both 9 kg conditions in the high PAL group and under all four conditions in the moderate PAL group. However, PAL and all PAL-related interactions were not significant in the integrated model, and direct comparisons of sex differences between PAL groups were also not significant. These findings suggest that the observed sex-related neuromuscular differences varied by lifting condition, whereas there was no statistical evidence that they were moderated by PAL. Accordingly, the PAL-stratified analyses should be regarded as exploratory subgroup analyses.

In the fNIRS analysis, significant sex-related differences after FDR correction were observed only in the moderate PAL group under the 3 kg weight-misprediction condition. Women showed higher task-related ΔHbO β-values than men across multiple prefrontal channels. This pattern may be consistent with greater prefrontal engagement during the processing of an unexpected lightweight load, in agreement with previous findings showing increased frontal activity during unpredictable weight changes [5]. However, attention and motor-planning demands were not measured directly in the present study. Therefore, alternative explanations, including relative task difficulty, muscle strength, movement strategy [11,12,13], and physiological characteristics, should also be considered [18]. The present findings should therefore be interpreted as a sex-related difference in task-related prefrontal hemodynamic responses observed under the 3 kg weight-misprediction condition rather than as evidence of a specific cognitive mechanism.

Because the analyses were based on standardized absolute loads of 3 and 9 kg, rather than individualized relative loads, differences in body size or strength may have contributed to individual variability in physiological responses. In addition, the subgroup and channel-wise analyses were based on relatively small sample sizes and were sufficiently powered only to detect very large effects. Therefore, these findings should be regarded as exploratory and require confirmation in larger and more balanced samples.

## 5. Conclusions

In the current study, normalized sEMG activity showed condition-dependent sex differences, whereas there was no statistical evidence that PAL moderated these differences, despite exploratory sex-related patterns within both PAL groups. Women in the moderate PAL group also showed higher task-related ΔHbO *β*-values than men during the 3 kg weight-misprediction condition. These preliminary findings suggest that task condition and sex may contribute to variability in neuromuscular and prefrontal hemodynamic responses during object lifting. Confirmation in larger and more balanced samples is required.

## Figures and Tables

**Table 1:** Integrated linear mixed-effects model results for normalized sEMG activity.

Effect	$\chi^{2}$	df	p
Condition	73.639	3	<0.001
Sex	14.559	1	<0.001
PAL	1.043	1	0.307
Condition × Sex	24.282	3	<0.001
Condition × PAL	2.299	3	0.513
Sex × PAL	0.847	1	0.357
Condition × Sex × PAL	0.418	3	0.937

Notes: Condition, sex, PAL, and their interactions were included as fixed effects. Participant-specific intercepts were included as random effects.

**Table 2:** Sex differences in ΔHbO β-values for the moderate PAL group under the 3 kg UKN wrong-guess condition. Only channels showing significant sex differences after FDR correction are reported.

Ch	t	df	q	Hedges’ g
03	−3.694	11.854	0.009	−1.755
04	−4.163	11.329	0.007	−2.082
05	−5.617	11.207	0.002	−2.614
06	−4.601	11.574	0.005	−2.283
07	−2.966	9.866	0.028	−1.536
09	−3.941	11.934	0.007	−1.922
10	−3.007	10.546	0.028	−1.534
11	−2.861	11.685	0.028	−1.414

Note: Negative t and Hedges’ g values indicate higher β-values in women than in men.

## Data Availability

The data that support the findings of this study are available from the corresponding author upon reasonable request.

## Supplementary Materials

The following supporting information can be downloaded at: https://www.mdpi.com/article/10.3390/bioengineering13080871/s1, Table S1: Channel-wise sex differences in task-related ΔHbO β-values across all four lifting conditions in the high and moderate PAL groups.
