# Supplementary material for: Condition-Dependent Sex Differences in Lumbar sEMG and Prefrontal Hemodynamics During Known-Weight and Weight-Misprediction Lifting: A Pilot Study
Source: Bioengineering (Basel). 2026 Jul 28;13(8):871. doi: 10.3390/bioengineering13080871 (PMC13509655; doi:10.3390/bioengineering13080871)
Supplement: Supplementary file 1 [file bioengineering-13-00871-s001.zip › bioengineering-4435354-supplementary.pdf]

Table S1. Channel-wise sex differences in task-related ΔHbO β-values across all four lifting conditions in the high and moderate PAL groups.

High PAL group

3 kg KNW					3 kg UKN wrong-guess				
**Ch**	t	**df**	p_{adj} **(FDR)**	**Hedges' g**	**Ch**	t	**df**	p_{adj} **(FDR)**	**Hedges' g**
01	-2.010	10.707	.232	-0.997	01	0.382	11.217	.744	0.188
02	-1.112	9.967	.367	-0.558	02	-0.357	8.730	.744	-0.183
03	-1.552	9.981	.284	-0.779	03	-0.760	11.226	.631	-0.374
04	-1.128	13.991	.367	-0.517	04	-0.547	13.622	.741	-0.257
05	-2.249	10.959	.232	-1.111	05	-1.328	10.222	.471	-0.664
06	-2.134	12.760	.232	-1.022	06	-2.412	12.849	.264	-1.154
07	-1.786	13.110	.243	-0.850	07	-0.850	11.065	.620	-0.419
08	-1.297	13.992	.359	-0.595	08	-2.187	10.328	.264	-1.091
09	-1.548	11.433	.284	-0.759	09	-1.615	10.602	.440	-0.802
10	-1.037	13.206	.367	-0.492	10	-2.535	7.376	.264	-1.335
11	-2.228	8.474	.232	-1.148	11	-1.609	7.941	.440	-0.838
12	-1.926	12.506	.232	-0.927	12	-1.041	10.377	.536	-0.519
13	-1.094	9.708	.367	-0.551	13	-1.318	9.078	.471	-0.672
14	-0.998	7.530	.374	-0.524	14	-1.082	6.432	.536	-0.583
15	-0.064	7.034	.951	-0.034	15	0.338	8.431	.744	0.174

9 kg KNW					9 kg UKN wrong-guess				
**Ch**	t	**df**	p_{adj} **(FDR)**	**Hedges' g**	**Ch**	t	**df**	p_{adj} **(FDR)**	**Hedges' g**
01	-0.597	13.741	.841	-0.270	01	-0.440	7.897	.775	-0.229
02	0.326	9.740	.867	0.164	02	0.001	7.715	1.000	0.000
03	0.470	12.939	.867	0.224	03	-1.119	12.928	.602	-0.534
04	0.367	12.617	.867	0.162	04	1.176	13.543	.602	0.529
05	0.164	11.904	.935	0.072	05	-0.752	13.955	.602	-0.344
06	-1.345	9.270	.455	-0.683	06	-0.833	13.999	.602	-0.383
07	-1.309	13.467	.455	-0.618	07	-1.660	13.993	.494	-0.761
08	-2.366	13.499	.212	-1.116	08	-2.089	13.988	.479	-0.958
09	-1.018	11.119	.620	-0.501	09	-0.276	13.458	.843	-0.124
10	-0.661	13.520	.841	-0.311	10	-1.046	12.719	.602	-0.502
11	-1.375	13.870	.455	-0.640	11	-0.885	13.784	.602	-0.413
12	-2.246	13.144	.212	-1.068	12	-0.727	11.941	.602	-0.353
13	-2.012	13.282	.244	-0.900	13	-1.613	12.449	.494	-0.711
14	-2.430	13.817	.212	-1.134	14	-2.034	12.472	.479	-0.980
15	-0.063	12.416	.951	-0.030	15	-0.751	10.321	.602	-0.375

Moderate PAL group

3 kg KNW					3 kg UKN wrong-guess				
**Ch**	***t***	**df**	***q***	**Hedges' g**	**Ch**	***t***	**df**	***q***	**Hedges' g**
01	-1.008	10.837	.419	-0.511	01	-0.857	6.695	.421	-0.478
02	-2.016	10.769	.174	-1.023	02	-1.160	6.828	.305	-0.645
03	-1.746	11.786	.174	-0.859	03	-3.694	11.854	**.009**	-1.755
04	-2.649	11.942	.174	-1.291	04	-4.163	11.329	**.007**	-2.082
05	-1.823	10.235	.174	-0.936	05	-5.617	11.207	**.002**	-2.614
06	-1.410	10.617	.255	-0.648	06	-4.601	11.574	**.005**	-2.283
07	-2.428	10.552	.174	-1.239	07	-2.966	9.866	**.028**	-1.536
08	0.093	6.928	.929	0.051	08	-1.758	11.412	.132	-0.877
09	-2.076	9.322	.174	-1.088	09	-3.941	11.934	**.007**	-1.922
10	-1.971	10.449	.174	-1.008	10	-3.007	10.546	**.028**	-1.534
11	-1.709	10.700	.174	-0.869	11	-2.861	11.685	**.028**	-1.414
12	-0.832	10.816	.488	-0.422	12	-2.547	8.318	.050	-1.365
13	-2.031	8.227	.174	-1.090	13	-2.556	9.092	.050	-1.145
14	-1.776	10.754	.174	-0.818	14	-1.463	12.000	.195	-0.706
15	-0.130	11.671	.929	-0.061	15	2.013	6.633	.118	1.125
**9 kg KNW**					**9 kg UKN wrong-guess**				
**Ch**	***t***	**df**	***q***	**Hedges' g**	**Ch**	***t***	**df**	***q***	**Hedges' g**
01	-0.753	11.213	.500	-0.350	01	-0.220	10.330	.830	-0.113
02	-1.478	11.915	.206	-0.705	02	-1.200	9.593	.519	-0.625
03	-2.094	10.404	.116	-1.072	03	-0.560	9.465	.679	-0.293
04	-2.908	11.774	.082	-1.377	04	-3.545	8.447	.104	-1.893
05	-2.552	11.264	.082	-1.279	05	-1.139	7.331	.519	-0.625
06	-1.779	8.688	.184	-0.945	06	-0.839	8.981	.635	-0.443
07	-2.851	11.970	.082	-1.366	07	-2.320	11.411	.199	-1.157
08	-2.309	11.160	.088	-1.160	08	-1.375	10.508	.519	-0.702
09	-1.677	8.242	.196	-0.900	09	-1.107	5.880	.519	-0.633
10	-2.593	9.030	.082	-1.367	10	-1.484	10.533	.519	-0.757
11	-1.548	9.612	.206	-0.806	11	-1.369	6.063	.519	-0.779
12	-0.872	8.761	.469	-0.462	12	-0.678	7.437	.648	-0.371
13	-2.726	7.557	.082	-1.487	13	-2.621	10.418	.186	-1.341
14	-2.435	11.233	.082	-1.221	14	-0.737	11.999	.648	-0.355
15	-0.059	10.095	.954	-0.030	15	0.452	10.585	.708	0.230

Notes: Sex comparisons were performed using Welch's independent-samples t-tests. FDR correction was applied separately across the 15 channels within each condition and PAL group. Bold q-values indicate $q<.05$. Negative t and Hedges' g values indicate higher β-values in

women than in men. KNW, known weight; UKN, unknown weight; PAL, physical activity level.
